# Exploring highly dispersive optical solitons and modulation instability in nonlinear Schrödinger equations with nonlocal self phase modulation and polarization dispersion

**DOI:** 10.1038/s41598-025-09710-8

**Published:** 2025-07-25

**Authors:** Wafy M. Hasan, Hamdy M. Ahmed, Ahmed M. Ahmed, Haytham M. Rezk, Wafaa B. Rabie

**Affiliations:** 1 Department of Mathematics, Faculty of Science, Al-Azhar University, Cairo, Egypt; 2 Department of Basic Sciences, Faculty of Engineering Technology, ElSewedy University of Technology, Cairo, Egypt; 3 Department of Physics and Engineering Mathematics, Higher Institute of Engineering, El Shorouk Academy, Cairo, Egypt; 4 Department of Basic Sciences, Higher Institute of Engineering and Technology, El-Bagour, Menoufia Egypt

**Keywords:** Extended NLSE, IMETFM, Optical soliton solutions, Soliton behavior visualization, Modulation instability, Applied mathematics, Computational science

## Abstract

This study explores the dynamics of highly dispersive optical solitons in nonlinear Schrödinger equations (NLSE) with non-local self-phase modulation (SPM) and polarization-mode dispersion (PMD). These nonlinear effects significantly influence soliton propagation and stability in advanced optical communication systems. Employing the Improved Modified Extended Tanh-Function Method (IMETFM), we derive exact soliton solutions, including bright, dark, singular, and combo solitons, under specific parametric conditions. The IMETFM effectively handles the complexity of the NLSE, incorporating higher-order dispersion terms (up to sixth-order) and non-local nonlinearities. Additionally, we perform a modulation instability (MI) analysis to examine the stability of steady-state solutions. This analysis uncovers the conditions under which instabilities emerge due to the interplay between dispersion and nonlinearity. The MI study offers critical insights into the growth of wave perturbations, thereby advancing the understanding of soliton stability dynamics. Graphical representations of the solutions illustrate their behavior, emphasizing the impact of non-local SPM, PMD, and MI on soliton dynamics. These findings offer valuable insights for optimizing high-capacity optical communication systems and fiber laser technologies, with broader implications for nonlinear wave phenomena in birefringent fibers and other nonlinear physical systems. This work advances the theoretical framework for soliton dynamics and lays a foundation for future experimental validations and practical applications in nonlinear optics.

## Introduction

Nonlinear partial differential equations (NLPDEs) are essential tools in understanding wave phenomena across various fields of science and engineering. These equations model systems where interactions between different variables or components are not proportional, leading to complex behaviors such as shock waves, solitons, and turbulence. The study of nonlinear PDEs has broad applications in areas ranging from fluid dynamics and acoustics to environmental science and material science. For instance, the Navier-Stokes equations, which govern fluid dynamics, are nonlinear and their solutions reveal critical insights into turbulence, wave breaking, and complex flow behavior^1,2^. The Korteweg-de Vries (KdV) equation, a nonlinear PDE, describes soliton solutions in water waves and has been foundational in the study of nonlinear wave propagation^3,4^. In environmental science, nonlinear PDEs are used to model wave propagation in the atmosphere and oceans, such as the nonlinear advection-diffusion equations that govern pollutant dispersion in air and water^5,6^. The impact of nonlinear wave phenomena on weather patterns, ocean currents, and climate dynamics is another area where these equations provide essential insights, particularly in the study of extreme weather events and their consequences for climate change modeling^7,8^. Furthermore, in engineering, nonlinear PDEs are employed to predict and control shock waves in aerodynamics, stress-strain relationships in materials under large deformations, and wave behavior in nonlinear optical fibers^9,10^. Thus, the study of nonlinear PDEs offers powerful tools for understanding and solving complex real-world problems across multiple disciplines.

A variety of techniques have been developed to find exact solutions to nonlinear evolution equations (NLEEs), such as the modified extended tanh-function method^11^, the extended simplest equation method^12,13^, the extended rational Sinh-Cosh and sine-Cosine methods^14^, the semi-inverse variational technique^15^, the extended Sinh-Gordon equation expansion^16^, the modified extended direct algebraic method^17,18^, the modified extended mapping method^19,20^, the modified Sardar Sub-Equation Method^21^, the extended F-Expansion method^22,23^, and the Improved Modified Extended Tanh-Function Method^24^. Other methods include the Homogeneous Balance Method^25^, Bäcklund Transformation, and Riccati-Bernoulli methods^26^. These methods are crucial for analyzing complex physical phenomena, including wave propagation, fluid dynamics, and thermal interactions, facilitating advancements in mathematics, physics, and engineering.

The study of nonlinear wave propagation in optical fibers plays a crucial role in modern photonic and telecommunication systems. Among the various nonlinear effects, self-phase modulation (SPM) is one of the most significant, as it governs the interplay between dispersion and nonlinearity in optical soliton dynamics^27^. The coupled SPM equations describe the evolution of optical solitons in birefringent fibers, where the impact of polarization-mode dispersion (PMD) and higher-order dispersive effects must be considered. Unlike Ref.^27^, which used alternative methods and could not retrieve dark solitons, our study employs IMETFM to derive a comprehensive set of solutions, including dark solitons, and includes modulation instability analysis to assess stability. This study focuses on single-soliton solutions and their coupled dynamics through cross-phase modulation (XPM), rather than multi-soliton collisions or interactions.

The nonlinear Schrödinger equation (NLSE) is a fundamental equation in nonlinear science, describing the evolution of wave packets in dispersive and nonlinear media. In the context of highly dispersive optical solitons, the governing model is a modified NLSE incorporating third-order (3OD), fourth-order (4OD), fifth-order (5OD), and sixth-order dispersion (6OD). The coupled system is:

1 $$\begin{aligned} & i u_t + i \alpha _1 u_x + \alpha _2 u_{xx} + i \alpha _3 u_{xxx} + \alpha _4 u_{xxxx} + i \alpha _5 u_{xxxxx} + \alpha _6 u_{xxxxxx} + \left( \beta _1 (|u|^2)_{xx} + \beta _2 (|v|^2)_{xx} \right) u = 0, \end{aligned}$$

2 $$\begin{aligned} & i v_t + i \gamma _1 v_x + \gamma _2 v_{xx} + i \gamma _3 v_{xxx} + \gamma _4 v_{xxxx} + i \gamma _5 v_{xxxxx} + \gamma _6 v_{xxxxxx} + \left( \lambda _1 (|v|^2)_{xx} + \lambda _2 (|u|^2)_{xx} \right) v = 0, \end{aligned}$$

where *u*(*x*, *t*) and *v*(*x*, *t*) represent the orthogonally polarized components, $\alpha _i$ and $\gamma _i$ (for $i = 1,2,3,4,5,6$) describe dispersion effects, and $\beta _j$ and $\lambda _j$ (for $j = 1,2$) represent SPM and XPM contributions. These equations are particularly relevant for the study of nonlocal nonlinear optics, where the response of the medium depends on the field’s spatial distribution. The incorporation of polarization-mode dispersion (PMD) introduces additional complexity, making the search for analytical and numerical solutions essential. Finding new wave solutions to these coupled SPM equations can lead to a deeper understanding of soliton interactions, stability, and potential applications in optical communication and fiber laser technology.

In Ref.^27^, the authors investigated highly dispersive optical solitons in birefringent fibers with polarization-mode dispersion (PMD) under non-local self-phase modulation (SPM), employing the Extended Simplest Equation Method and Kudryashov’s Approach. While their work successfully derived bright and singular soliton solutions for the higher-order (3OD-6OD) nonlinear Schrödinger equation (NLSE), it failed to obtain other types of solutions, exposing a key constraint in current analytical techniques. This study is motivated by the critical role of optical solitons in modern high-capacity communication systems and fiber laser technologies, where the interplay between non-local SPM and PMD introduces complex dynamics that remain poorly understood. While prior research^28–31^ has advanced the field–including investigations into generalized NLSE models and non-local effects–significant gaps persist in obtaining a complete spectrum of soliton solutions and characterizing their stability. To address these challenges, we adopt the Improved Modified Extended Tanh-Function Method (IMETFM) to achieve three key advances: derivation of a comprehensive set of soliton solutions, including bright, dark, singular, and combined types. Development of additional solutions encompassing hyperbolic, Jacobi elliptic, periodic, rational, and exponential forms. Systematic modulation instability analysis for stability evaluation. Our approach not only overcomes prior methodological limitations but also provides practical tools for optimizing photonic systems. The results offer new theoretical insights into nonlinear wave propagation and establish testable predictions for experiments in fiber optics, ultrafast lasers, and related fields. By expanding the mathematical framework for non-local effects in dispersive media, this work contributes directly to the advancement of next-generation optical technologies.

This research is organized into six main sections. The first “Introduction” provides an introduction, setting the context and objectives. The second section “IMETFM methodology” discusses the IMETF method, followed by a detailed explanation of exact solutions for the coupled SPM model in the third section “Exact solutions for coupled SPM model”. The fourth section “Modulation instability analysis” discusses the modulation instability of the studied system. The fifth section “Graphical overview” presents graphical representations to visualize key findings. The sixth section “Results and discussions” discusses the findings, and the seventh and final section “Conclusion” concludes with a summary and implications of the research.

## IMETFM methodology

Here, we outline the primary steps of the IMETF method^24^ as follows:

Consider the NLPDE:

3 $$\begin{aligned} F(z, z_{t}, z_{x}, z_{tt}, z_{xx}, \, \dots \dots ) = 0, \end{aligned}$$

where $z = z(x, t)$ denotes an unknown function, and *F* is a polynomial that includes *z* and its partial derivatives concerning *t* and *x* and involves the highest-order derivatives and nonlinear terms.

*Step I:* To simplify Eq. (3), we introduce the following traveling wave transformation:

4 $$\begin{aligned} z(x, t) = \Upsilon (\xi ), \quad \quad \xi = a_{1} \, x + a_{2} \, t, \end{aligned}$$

where $a_{1}$ and $a_{2}$ are constants that will be determined later. Substituting this transformation into Eq. (3) reduces it to a nonlinear ordinary differential equation (ODE):

5 $$\begin{aligned} G(\Upsilon , \Upsilon ', \Upsilon '', \, \dots \dots ) = 0. \end{aligned}$$

*Step II:* Next, we propose that the solution to Eq. (5) takes the form:

6 $$\begin{aligned} \Upsilon (\xi ) = \sum _{i=0}^{N} r_{i} \, W^{i} + \sum _{i=1}^{N} s_{i} \, W^{-i}, \end{aligned}$$

where $r_{i}$ and $s_{i}$ are constants, *W* is a function that satisfies the following:

7 $$\begin{aligned} W' = \epsilon \sqrt{c_0 + c_1 W + c_2 W^2 + c_3 W^3 + c_4 W^4}, \end{aligned}$$

with $\epsilon = \pm 1$ and $c_i$ $(i= 0, 1, 2, 3, 4)$ representing real-valued constants. This equation offers numerous fundamental solutions that can be used to derive further exact solutions for Eq. (3).

*Step III:* The value of *N* in Eq. (6) is determined by balancing the highest-order derivative and the non-linear term present in Eq. (5).

*Step IV:* Substituting Eq. (6) into Eq. (5) while incorporating Eq. (7) generates a polynomial in terms of *W*. By grouping coefficients of like powers and setting them to *zero*, we create a system of equations. These can be solved utilizing mathematical tools such as *Maple*, *MATLAB*, or *Wolfram Mathematica* to evaluate the values of the unknown parameters $a_{1}, a_{2}, r_{0}, r_{i},$ and $s_{i}$ ($i= 1, 2, 3, \, \dots$). Consequently, this process yields the exact solutions for Eq. (3).

## Exact solutions for coupled SPM model

Here, we employ the IMETF method to derive exact solutions for Eqs. (1) and (2). The solutions are assumed to have the following form:

8 $$\begin{aligned} \begin{aligned} u(x, t)&= U(\xi ) \exp [i (- k x + \omega t)], \\ v(x, t)&= V(\xi ) \exp [i (- k x + \omega t)], \end{aligned} \end{aligned}$$

and

9 $$\begin{aligned} \xi&= x - c \, t , \end{aligned}$$

where *c*, *k*, and $\omega$ are non-zero constants, where *c* represents the soliton’s velocity, *k* denotes its wave number, and $\omega$ corresponds to its frequency. We will commence by implementing the wave transformations indicated in Eqs. (8) to Eqs. (1) and (2), then separating the real and imaginary components to get the following equations:

10 $$\begin{aligned} \nonumber \mathrm {Re_1: \quad }&\alpha _6 U^{(6)}(\xi ) + (\alpha _4 + 5k \alpha _5 - 15k^2 \alpha _6) U^{(4)}(\xi ) + (\alpha _2 + 3k \alpha _3 - 6k^2 \alpha _4 - 10k^3 \alpha _5 + 15k^4 \alpha _6) U^{\prime \prime }(\xi ) \\ \nonumber&+ 2\beta _1 \left( U(\xi )\right) ^2 U^{\prime \prime }(\xi ) + 2\beta _2 U(\xi ) V(\xi ) V^{\prime \prime }(\xi ) + 2\beta _1 U(\xi ) \left( U^{\prime }(\xi ) \right) ^2 + 2\beta _2 U(\xi ) \left( V^{\prime }(\xi ) \right) ^2 \\&+ \left( -\omega + k\alpha _1 - k^2\alpha _2 - k^3\alpha _3 + k^4\alpha _4 + k^5\alpha _5 - k^6\alpha _6\right) U(\xi ) = 0. & \end{aligned}$$

11 $$\begin{aligned} \nonumber \mathrm {Re_2: \quad }&\gamma _6 V^{(6)}(\xi ) + \left( \gamma _4 + 5k \gamma _5 - 15k^2 \gamma _6\right) V^{(4)}(\xi ) + \left( \gamma _2 + 3k \gamma _3 - 6k^2 \gamma _4 - 10k^3 \gamma _5 + 15k^4 \gamma _6\right) V^{\prime \prime }(\xi ) \\ \nonumber&+ 2\lambda _2 U(\xi ) V(\xi ) U^{\prime \prime }(\xi ) + 2\lambda _1 \left( V(\xi )\right) ^2 V^{\prime \prime }(\xi ) + 2\lambda _2 V(\xi ) \left( U^{\prime }(\xi ) \right) ^2 + 2\lambda _1 V(\xi ) \left( V^{\prime }(\xi ) \right) ^2 \\&+ \left( -\omega + k\gamma _1 - k^2\gamma _2 - k^3\gamma _3 + k^4\gamma _4 + k^5\gamma _5 - k^6\gamma _6\right) V(\xi ) = 0. & \end{aligned}$$

12 $$\begin{aligned} \nonumber \mathrm {Im_1: \quad }&\left( -\alpha _5 + 6k \alpha _6\right) U^{(5)}(\xi ) + \left( -\alpha _3 + 4k \alpha _4 + 10k^2 \alpha _5 - 20k^3 \alpha _6\right) U^{\prime \prime \prime }(\xi ) \\&+ \left( c - \alpha _1 + 2k \alpha _2 + 3k^2 \alpha _3 - 4k^3 \alpha _4 - 5k^4 \alpha _5 + 6k^5 \alpha _6\right) U^{\prime }(\xi ) = 0. & \end{aligned}$$

13 $$\begin{aligned} \nonumber \mathrm {Im_2: \quad }&(-\gamma _5 + 6k \gamma _6) V^{(5)}(\xi ) + (-\gamma _3 + 4k \gamma _4 + 10k^2 \gamma _5 - 20k^3 \gamma _6) V^{\prime \prime \prime }(\xi ) \\&+ (c - \gamma _1 + 2k \gamma _2 + 3k^2 \gamma _3 - 4k^3 \gamma _4 - 5k^4 \gamma _5 + 6k^5 \gamma _6) V^{\prime }(\xi ) = 0. & \end{aligned}$$

Let

14 $$\begin{aligned} V(\xi ) = A \, U(\xi ), \end{aligned}$$

provided $A\ne \{0, 1\}$. Now, we can represent Eqs. (10)–(13) as follows:

15 $$\begin{aligned} \nonumber \mathrm {Re_1: \quad }&\alpha _6 U^{(6)}(\xi ) + \left( \alpha _4 + 5k \alpha _5 - 15k^2 \alpha _6\right) U^{(4)}(\xi ) + \left( \alpha _2 + 3 k \alpha _3 - 6 k^2 \alpha _4 - 10 k^3 \alpha _5 + 15 k^4 \alpha _6 \right) U^{\prime \prime }(\xi ) \\ \nonumber&+ 2 \left( \beta _1 + A^2 \beta _2\right) \left( U(\xi )\right) ^2 U^{\prime \prime }(\xi ) + 2 \left( \beta _1 + A^2 \beta _2\right) U(\xi ) \left( U^{\prime }(\xi )\right) ^2 \\&+ \left( -\omega + k \alpha _1 - k^2 \alpha _2 - k^3 \alpha _3 + k^4 \alpha _4 + k^5 \alpha _5 - k^6 \alpha _6\right) U(\xi ) = 0. & \end{aligned}$$

16 $$\begin{aligned} \nonumber \mathrm {Re_2: \quad }&A \gamma _6 U^{(6)}(\xi ) + A \left( \gamma _4 + 5k \gamma _5 - 15k^2 \gamma _6\right) U^{(4)}(\xi ) + A \left( \gamma _2 + 3 k \gamma _3 - 6 k^2 \gamma _4 - 10 k^3 \gamma _5 + 15 k^4 \gamma _6\right) U^{\prime \prime }(\xi ) \\ \nonumber&+ 2 A \left( A^2 \lambda _1 + \lambda _2\right) (U(\xi ))^2 U^{\prime \prime }(\xi ) + 2 A \left( A^2 \lambda _1 + \lambda _2\right) U(\xi ) \left( U^{\prime }(\xi )\right) ^2 \\&+ A \left( -\omega + k \gamma _1 - k^2 \gamma _2 - k^3 \gamma _3 + k^4 \gamma _4 + k^5 \gamma _5 - k^6 \gamma _6\right) U(\xi ) = 0. & \end{aligned}$$

17 $$\begin{aligned} \nonumber \mathrm {Im_1: \quad }&\left( -\alpha _5 + 6k \alpha _6\right) U^{(5)}(\xi ) + \left( -\alpha _3 + 4k \alpha _4 + 10k^2 \alpha _5 - 20k^3 \alpha _6\right) U^{\prime \prime \prime }(\xi ) \\&+ \left( c - \alpha _1 + 2k \alpha _2 + 3k^2 \alpha _3 - 4k^3 \alpha _4 - 5k^4 \alpha _5 + 6k^5 \alpha _6\right) U^{\prime }(\xi ) = 0. & \end{aligned}$$

18 $$\begin{aligned} \nonumber \mathrm {Im_2: \quad }&A \left( -\gamma _5 + 6k \gamma _6\right) U^{(5)}(\xi ) + A \left( -\gamma _3 + 4k \gamma _4 + 10k^2 \gamma _5 - 20k^3 \gamma _6\right) U^{\prime \prime \prime }(\xi ) \\&+ A \left( c - \gamma _1 + 2k \gamma _2 + 3k^2 \gamma _3 - 4k^3 \gamma _4 - 5k^4 \gamma _5 + 6k^5 \gamma _6\right) U^{\prime }(\xi ) = 0. & \end{aligned}$$

Setting the coefficients of the linearly independent functions in Eqs. (17) and (18) to zero gives us:

19 $$\begin{aligned} k= & \frac{\alpha _5}{6\alpha _6} = \frac{\gamma _5}{6\gamma _6}, \end{aligned}$$

20 $$\begin{aligned} c= & \alpha _1 - 2k \alpha _2 - 3k^2 \alpha _3 + 4k^3 \alpha _4 + 5k^4 \alpha _5 - 6k^5 \alpha _6 = \gamma _1 - 2k \gamma _2 - 3k^2 \gamma _3 + 4k^3 \gamma _4 + 5k^4 \gamma _5 - 6k^5 \gamma _6, \end{aligned}$$

21 $$\begin{aligned} & \alpha _3 - 4k \alpha _4 - 10k^2 \alpha _5 + 20k^3 \alpha _6 = \gamma _3 - 4k \gamma _4 - 10k^2 \gamma _5 + 20k^3 \gamma _6 = 0. \end{aligned}$$

Through Eqs. (15) and (16), we notice that identical forms, under the following constraint conditions:

22 $$\begin{aligned} \begin{aligned}&\alpha _6 = \gamma _6, \\&\alpha _4 + 5k \alpha _5 - 15k^2 \alpha _6 = \gamma _4 + 5k \gamma _5 - 15k^2 \gamma _6, \\&\alpha _2 + 3k \alpha _3 - 6k^2 \alpha _4 - 10k^3 \alpha _5 + 15k^4 \alpha _6 = \gamma _2 + 3k \gamma _3 - 6k^2 \gamma _4 - 10k^3 \gamma _5 + 15k^4 \gamma _6, \\&\omega - k \alpha _1 + k^2 \alpha _2 + k^3 \alpha _3 - k^4 \alpha _4 - k^5 \alpha _5 + k^6 \alpha _6 = \omega - k \gamma _1 + k^2 \gamma _2 + k^3 \gamma _3 - k^4 \gamma _4 - k^5 \gamma _5 + k^6 \gamma _6, \\&2 (\beta _1 + A^2 \beta _2) = 2 (A^2 \lambda _1 + \lambda _2). \end{aligned} & \Bigg \} \end{aligned}$$

From these conditions (22) we can simply conclude:

23 $$\begin{aligned}&A = \sqrt{\frac{\lambda _2 - \beta _1}{\beta _2 - \lambda _1}}, \;\; \text {where} \; (\lambda _2 - \beta _1) (\beta _2 - \lambda _1) > 0 \; \text {and} \; (\lambda _2 - \beta _1) \ne (\beta _2 - \lambda _1). & \end{aligned}$$

Now, we can now express Eq. (15) as follows:

24 $$\begin{aligned} U^{(6)}(\xi ) + \sigma _1 U^{(4)}(\xi ) + \sigma _2 U;'(\xi ) + \sigma _3 U(\xi ) + \sigma _4 \left( U(\xi ) \left( U'(\xi ) \right) ^2 + \left( U(\xi )\right) ^2 U;;'(\xi ) \right) = 0. \end{aligned}$$

where

25 $$\begin{aligned} \begin{aligned}&\sigma _1 = \frac{1}{\alpha _6} \left( \alpha _4 + 5k \alpha _5 - 15k^2 \alpha _6 \right) , \quad \sigma _2 = \frac{1}{\alpha _6} \left( \alpha _2 + 3k \alpha _3 - 6k^2 \alpha _4 - 10k^3 \alpha _5 + 15k^4 \alpha _6 \right) , \\&\sigma _3 = \frac{1}{\alpha _6} \left( -\omega + k \alpha _1 - k^2 \alpha _2 - k^3 \alpha _3 + k^4 \alpha _4 + k^5 \alpha _5 - k^6 \alpha _6 \right) , \quad \sigma _4 = \frac{2}{\alpha _6} \left( \beta _1 + A^2 \beta _2 \right) . \end{aligned} & \end{aligned}$$

To determine the form of the solution, the equilibrium principle is applied to Eq. (24), indicating that $N=2$. Consequently, based on Eq. (6), the solution for $U(\xi )$ is expressed as follows:

26 $$\begin{aligned} U(\xi ) = r_{0} + r_{1} \, W(\xi ) + r_{2} \, W(\xi )^{2} + s_{1} \, W(\xi )^{-1} + s_{2} \, W(\xi )^{-2}, \end{aligned}$$

where $r_{0}, r_{1}, r_{2}, s_{1}$, and $s_{2}$ are constants to be determined.

Substituting the expression for $U(\xi )$ from Eq. (26) and incorporating Eq. (7) into Eq. (24), we collect the coefficients of powers of $W^{i}$ for $i \in [-8, 8]$. Setting these coefficients to *zero* creates a system of equations which, when solved, provides the exact solutions to the Eqs. (1) and (2) as follows:

**Case Study 1:** Suppose that $c_0 = c_1 = c_3 = 0$. In this case, we obtain the following solution sets:

$$\begin{aligned} {\textbf {[1.1]}}\, r_0= & \frac{24 \sqrt{\frac{2}{7}} \, c_2}{\sqrt{-\sigma _4}}, \quad r_1 = 0, \quad r_2 = \frac{6 \sqrt{14} \, c_4}{\sqrt{-\sigma _4}}, \quad s_1 = s_2 = 0, \quad \sigma _1 = 16 \, c_2, \quad \sigma _2 = \frac{592 \, c_2^2}{7}, \quad \sigma _3 = 0. \\ {\textbf {[1.2]}}\, r_0= & r_1 = 0, \quad r_2 = \frac{6 \sqrt{14} \, c_4}{\sqrt{-\sigma _4}}, \quad s_1 = s_2 = 0, \quad \sigma _1 = -\frac{112 \, c_2}{5}, \quad \sigma _2 = 112 \, c_2^2, \quad \sigma _3 = -\frac{768 \, c_2^3}{5}. \end{aligned}$$

Based on [1.1], the solutions to Eqs. (1) and (2) below are as follows:

${\textbf {[1.1.1]}}$ When $c_4, \sigma _4 < 0$ and $c_2 > 0$, the solutions are:

27 $$\begin{aligned} u_{1.1.1}(x, t)= & - 6 c_2 \sqrt{\frac{-2}{7 \sigma _4}} \, \left( -4 + 7 {{\,\textrm{sech}\,}}^2[(x - ct) \sqrt{c_2}\,]\right) e^{i(- k x + t \omega )}, \end{aligned}$$

28 $$\begin{aligned} v_{1.1.1}(x, t)= & - 6 c_2 A \sqrt{\frac{-2}{7 \sigma _4}} \, \left( -4 + 7 {{\,\textrm{sech}\,}}^2[(x - ct) \sqrt{c_2}\,]\right) e^{i(- k x + t \omega )}, \end{aligned}$$

these are referred to as bright soliton solutions.

${\textbf {[1.1.2]}}$ When $c_4 > 0$, $\sigma _4 < 0$ and $c_2 < 0$, the solutions become:

29 $$\begin{aligned} u_{1.1.2}(x, t)= & - 6 c_2 \sqrt{\frac{-2}{7 \sigma _4}} \, \left( -4 + 7 \sec ^2[(x - ct) \sqrt{-c_2}\,]\right) e^{i(- k x + t \omega )}, \end{aligned}$$

30 $$\begin{aligned} v_{1.1.2}(x, t)= & - 6 c_2 A \sqrt{\frac{-2}{7 \sigma _4}} \, \left( -4 + 7 \sec ^2[(x - ct) \sqrt{-c_2}\,]\right) e^{i(- k x + t \omega )}, \end{aligned}$$

these are referred to as singular periodic solutions.

${\textbf {[1.1.3]}}$ When $c_4 > 0$, $\sigma _4 < 0$ and $c_2 = 0$, the resulting solutions are:

31 $$\begin{aligned} u_{1.1.3}(x, t)= & \frac{6 \sqrt{14} }{(x - ct)^2 \, \sqrt{-\sigma _4}} \, e^{i (-k x + t \omega )}, \end{aligned}$$

32 $$\begin{aligned} v_{1.1.3}(x, t)= & \frac{6 \sqrt{14} \, A}{(x - ct)^2 \, \sqrt{-\sigma _4}} \, e^{i (-k x + t \omega )}, \end{aligned}$$

these are referred to as rational solutions.

Based on [1.2], the solutions to Eqs. (1) and (2) below are as follows:

${\textbf {[1.2.1]}}$ When $c_4, \sigma _4 < 0$ and $c_2 > 0$, the solutions are:

33 $$\begin{aligned} u_{1.2.1}(x, t)= & - 6 c_2 \sqrt{\frac{-14}{ \sigma _4}} \, {{\,\textrm{sech}\,}}^2[(x - ct) \sqrt{c_2}\,] \, e^{i(- k x + t \omega )}, \end{aligned}$$

34 $$\begin{aligned} v_{1.2.1}(x, t)= & - 6 c_2 A \sqrt{\frac{-14}{ \sigma _4}} \, {{\,\textrm{sech}\,}}^2[(x - ct) \sqrt{c_2}\,] \, e^{i(- k x + t \omega )}, \end{aligned}$$

these are referred to as bright soliton solutions.

${\textbf {[1.2.2]}}$ When $c_4 > 0$, $\sigma _4 < 0$ and $c_2 < 0$, the solutions become:

35 $$\begin{aligned} u_{1.2.2}(x, t)= & - 6 c_2 \sqrt{\frac{-14}{ \sigma _4}} \, \sec ^2[(x - ct) \sqrt{-c_2}\,] \, e^{i(- k x + t \omega )}, \end{aligned}$$

36 $$\begin{aligned} v_{1.2.2}(x, t)= & - 6 c_2 A \sqrt{\frac{-14}{ \sigma _4}} \, \sec ^2[(x - ct) \sqrt{-c_2}\,] \, e^{i(- k x + t \omega )}, \end{aligned}$$

these are referred to as singular periodic solutions.

${\textbf {[1.2.3]}}$ When $c_4 > 0$, $\sigma _4 < 0$ and $c_2 = 0$, the resulting solutions are:

37 $$\begin{aligned} u_{1.2.3}(x, t)= & \frac{6 \sqrt{14} }{(x - ct)^2 \, \sqrt{-\sigma _4}} \, e^{i (-k x + t \omega )}, \end{aligned}$$

38 $$\begin{aligned} v_{1.2.3}(x, t)= & \frac{6 \sqrt{14} \, A}{(x - ct)^2 \, \sqrt{-\sigma _4}} \, e^{i (-k x + t \omega )}, \end{aligned}$$

these are referred to as rational solutions.

**Case Study 2:** If $c_1 = c_3 = 0$, we obtain the following solution sets:

$${\textbf {[2.1]}}$$ $$r_0 = \frac{3 \sqrt{14} c_2}{\sqrt{-\sigma _4}}, \quad r_2 = \frac{6 \sqrt{14} c_4}{\sqrt{-\sigma _4}}, \quad r_1 = s_1 = s_2 = 0, \quad \sigma _1 = \frac{56 c_2}{5}, \quad \sigma _2 = 28 c_2^2, \quad \sigma _3 = \frac{96 c_2^3}{5}, \quad c_0 = \frac{c_2^2}{4c_4}.$$

$${\textbf {[2.2]}}$$ $$r_0 = \frac{3 \sqrt{14} c_2}{\sqrt{-\sigma _4}}, \quad r_1 = r_2 = s_1 = 0, \quad s_2 = \frac{3 \sqrt{\frac{7}{2}} c_2^2}{c_4 \sqrt{-\sigma _4}}, \quad \sigma _1 = \frac{56 c_2}{5}, \quad \sigma _2 = 28 c_2^2, \quad \sigma _3 = \frac{96 c_2^3}{5}, \quad c_0 = \frac{c_2^2}{4c_4}.$$

$${\textbf {[2.3]}}$$ $$r_0 = r_1 = s_1 = 0, \; r_2 = \frac{6 \sqrt{14} c_4}{\sqrt{-\sigma _4}}, \quad s_2 = \frac{3 \sqrt{\frac{7}{2}} c_2^2}{c_4 \sqrt{-\sigma _4}}, \quad \sigma _1 = \frac{-112 c_2}{5}, \quad \sigma _2 = 352 c_2^2, \quad \sigma _3 = \frac{768 c_2^3}{5}, \quad c_0 = \frac{c_2^2}{4c_4}.$$

$${\textbf {[2.4]}}$$ $$r_0 = \frac{6 \sqrt{14} c_2}{\sqrt{-\sigma _4}}, \, r_1 = s_1 = 0, \, r_2 = \frac{6 \sqrt{14} c_4}{\sqrt{-\sigma _4}}, \, s_2 = \frac{3 \sqrt{\frac{7}{2}} c_2^2}{c_4 \sqrt{-\sigma _4}}, \, \sigma _1 = \frac{224 c_2}{5}, \, \sigma _2 = 448 c_2^2, \, \sigma _3 = \frac{6144 c_2^3}{5}, \, c_0 = \frac{c_2^2}{4c_4}.$$

$${\textbf {[2.5]}}$$ $$r_0 = r_1 = s_1 = 0 ,\, r_2 = \frac{6 \sqrt{14} c_4}{\sqrt{-\sigma _4}}, \, s_2 = \frac{6 \sqrt{14} c_0}{\sqrt{-\sigma _4}}, \, \sigma _1 = -\frac{112 c_2}{5}, \; \sigma _2 = 112 \left( c_2^2 + 6 c_0 \, c_4\right) , \; \sigma _3 = \frac{768}{5} \left( -c_2^3 + 8 c_0 \, c_2 \, c_4\right) .$$

Based on [2.1], the solutions to Eqs. (1) and (2) below are as follows:

${\textbf {[2.1.1]}}$ If $c_4>0, c_2, \sigma _4 <0,$ and $c_0=\frac{c_2^2}{4c_4}$, the resulting solutions are:

39 $$\begin{aligned} u_{2.1.1}(x, t)= & 3 c_2 \sqrt{\frac{-14}{ \sigma _4}} \, {{\,\textrm{sech}\,}}^2\left[ (x - ct) \sqrt{\frac{-c_2}{2}}\right] \, e^{i(- k x + t \omega )}, \end{aligned}$$

40 $$\begin{aligned} v_{2.1.1}(x, t)= & 3 c_2 A \sqrt{\frac{-14}{ \sigma _4}} \, {{\,\textrm{sech}\,}}^2\left[ (x - ct) \sqrt{\frac{-c_2}{2}}\right] \, e^{i(- k x + t \omega )}, \end{aligned}$$

these are referred to as bright soliton solutions.

${\textbf {[2.1.2]}}$ If $c_4>0, c_2>0, \sigma _4 < 0$ and $c_0=\frac{c_2^2}{4c_4}$, the resulting solutions are:

41 $$\begin{aligned} u_{2.1.2}(x, t)= & 3 c_2 \sqrt{\frac{-14}{ \sigma _4}} \, \sec ^2\left[ (x - ct) \sqrt{\frac{c_2}{2}}\right] \, e^{i(- k x + t \omega )}, \end{aligned}$$

42 $$\begin{aligned} v_{2.1.2}(x, t)= & 3 c_2 A \sqrt{\frac{-14}{ \sigma _4}} \, \sec ^2\left[ (x - ct) \sqrt{\frac{c_2}{2}}\right] \, e^{i(- k x + t \omega )}, \end{aligned}$$

these are referred to as singular periodic solutions.

While [2.2], the results derived are shown below:

${\textbf {[2.2.1]}}$ If $c_4>0, c_2, \sigma _4 <0,$ and $c_0=\frac{c_2^2}{4c_4}$, the resulting solutions are:

43 $$\begin{aligned} u_{2.2.1}(x, t)= & -3 c_2 \sqrt{\frac{-14}{ \sigma _4}} \, {{\,\textrm{csch}\,}}^2\left[ (x - ct) \sqrt{\frac{-c_2}{2}}\right] \, e^{i(- k x + t \omega )}, \end{aligned}$$

44 $$\begin{aligned} v_{2.2.1}(x, t)= & -3 c_2 A \sqrt{\frac{-14}{ \sigma _4}} \, {{\,\textrm{csch}\,}}^2\left[ (x - ct) \sqrt{\frac{-c_2}{2}}\right] \, e^{i(- k x + t \omega )}, \end{aligned}$$

these are referred to as singular soliton solutions.

${\textbf {[2.2.2]}}$ If $c_4>0, c_2>0, \sigma _4 < 0$ and $c_0=\frac{c_2^2}{4c_4}$, the resulting solutions are:

45 $$\begin{aligned} u_{2.2.2}(x, t)= & 3 c_2 \sqrt{\frac{-14}{ \sigma _4}} \, \csc ^2\left[ (x - ct) \sqrt{\frac{c_2}{2}}\right] \, e^{i(- k x + t \omega )}, \end{aligned}$$

46 $$\begin{aligned} v_{2.2.2}(x, t)= & 3 c_2 A \sqrt{\frac{-14}{ \sigma _4}} \, \csc ^2\left[ (x - ct) \sqrt{\frac{c_2}{2}}\right] \, e^{i(- k x + t \omega )}, \end{aligned}$$

these are referred to as singular periodic solutions.

And [2.3], the results derived are:

${\textbf {[2.3.1]}}$ If $c_4>0, c_2, \sigma _4 <0,$ and $c_0=\frac{c_2^2}{4c_4}$, the resulting solutions are:

47 $$\begin{aligned} u_{2.3.1}(x, t)= & -3 c_2 \sqrt{\frac{-14}{ \sigma _4}} \tanh ^2[(x - ct) \sqrt{\frac{-c_2}{2}}\, ] \left( 1 + \coth ^4[ (x - ct) \sqrt{\frac{-c_2}{2}}\, ]\right) \, e^{i(- k x + t \omega )}, \end{aligned}$$

48 $$\begin{aligned} v_{2.3.1}(x, t)= & -3 c_2 A \sqrt{\frac{-14}{ \sigma _4}} \tanh ^2[(x - ct) \sqrt{\frac{-c_2}{2}}\, ] \left( 1 + \coth ^4[ (x - ct) \sqrt{\frac{-c_2}{2}}\, ]\right) \, e^{i(- k x + t \omega )}, \end{aligned}$$

these are referred to as combo dark-singular soliton solutions.

${\textbf {[2.3.2]}}$ If $c_4>0, c_2>0, \sigma _4 < 0$ and $c_0=\frac{c_2^2}{4c_4}$, the resulting solutions are:

49 $$\begin{aligned} u_{2.3.2}(x, t)= & 3 c_2 \sqrt{\frac{-14}{ \sigma _4}} \tan ^2[(x - ct) \sqrt{\frac{c_2}{2}}\, ] \left( 1 + \cot ^4[ (x - ct) \sqrt{\frac{c_2}{2}}\, ]\right) \, e^{i(- k x + t \omega )}, \end{aligned}$$

50 $$\begin{aligned} v_{2.3.2}(x, t)= & 3 c_2 A \sqrt{\frac{-14}{ \sigma _4}} \tan ^2[(x - ct) \sqrt{\frac{c_2}{2}}\, ] \left( 1 + \cot ^4[ (x - ct) \sqrt{\frac{c_2}{2}}\, ]\right) \, e^{i(- k x + t \omega )}, \end{aligned}$$

these are referred to as singular periodic solutions.

And [2.4], the results derived are:

${\textbf {[2.4.1]}}$ If $c_4>0, c_2, \sigma _4 <0,$ and $c_0=\frac{c_2^2}{4c_4}$, the resulting solutions are:

51 $$\begin{aligned} u_{2.4.1}(x, t)= & -12 c_2 \sqrt{\frac{-14}{ \sigma _4}} \, {{\,\textrm{csch}\,}}^2[(x - ct) \sqrt{-2 c_2} \,] \, e^{i(- k x + t \omega )}, \end{aligned}$$

52 $$\begin{aligned} v_{2.4.1}(x, t)= & -12 A c_2 \sqrt{\frac{-14}{ \sigma _4}} \, {{\,\textrm{csch}\,}}^2[(x - ct) \sqrt{-2 c_2} \,] \, e^{i(- k x + t \omega )}, \end{aligned}$$

these are referred to as singular soliton solutions.

${\textbf {[2.4.2]}}$ If $c_4>0, \, c_2, \sigma _4 < 0$ and $c_0=\frac{c_2^2}{4c_4}$, the resulting solutions are:

53 $$\begin{aligned} u_{2.4.2}(x, t)= & 12 c_2 \sqrt{\frac{-14}{ \sigma _4}} \, \csc ^2[(x - ct) \sqrt{-2 c_2} \,] \, e^{i(- k x + t \omega )}, \end{aligned}$$

54 $$\begin{aligned} v_{2.4.2}(x, t)= & 12 c_2 A \sqrt{\frac{-14}{ \sigma _4}} \, \csc ^2[(x - ct) \sqrt{-2 c_2} \,] \, e^{i(- k x + t \omega )}, \end{aligned}$$

these are referred to as singular periodic solutions.

But [2.5], the results derived are shown below:

${\textbf {[2.5.1]}}$ If $c_4 , \sigma _4 <0, c_2>0, c_0 = \frac{{c_2}^2 m^2 (1 - m^2)}{c_4 (2 m^2 - 1)^2}$, and $\frac{1}{\sqrt{2}}<m\le 1$, the resulting solutions are:

55 $$\begin{aligned} u_{2.5.1}(x, t)= & -\frac{6 c_2 \, \sqrt{14} \left( 1 - m^2 + m^2 \, {{\,\textrm{cn}\,}}^4 \left[ (x - c t) \sqrt{\frac{c_2}{2 m^2 - 1}} \right] \right) }{\left( 2 m^2 - 1 \right) \sqrt{-\sigma _4} \, {{\,\textrm{cn}\,}}^2 \left[ (x - c t) \sqrt{\frac{c_2}{2 m^2 - 1}} \right] } \, e^{i(- k x + t \omega )}, \end{aligned}$$

56 $$\begin{aligned} v_{2.5.1}(x, t)= & -\frac{6 c_2 \, A \, \sqrt{14} \left( 1 - m^2 + m^2 \, {{\,\textrm{cn}\,}}^4 \left[ (x - c t) \sqrt{\frac{c_2}{2 m^2 - 1}} \right] \right) }{\left( 2 m^2 - 1 \right) \sqrt{-\sigma _4} \, {{\,\textrm{cn}\,}}^2 \left[ (x - c t) \sqrt{\frac{c_2}{2 m^2 - 1}} \right] } \, e^{i(- k x + t \omega )}, \end{aligned}$$

these are referred to as Jacobi elliptic solutions.

For $m=1$, bright soliton-type solutions are offered as follows:

57 $$\begin{aligned} u_{2.5.1a}(x, t)= & -6 c_2 \sqrt{\frac{-14}{ \sigma _4}} \, {{\,\textrm{sech}\,}}^2[(x - ct) \sqrt{c_2}\,] \, e^{i(- k x + t \omega )}, \end{aligned}$$

58 $$\begin{aligned} v_{2.5.1a}(x, t)= & -6 c_2 A \sqrt{\frac{-14}{ \sigma _4}} \, {{\,\textrm{sech}\,}}^2[(x - ct) \sqrt{c_2}\,] \, e^{i(- k x + t \omega )}. \end{aligned}$$

${\textbf {[2.5.2]}}$ If $c_4 , \sigma _4 <0, c_2>0, c_0 = \frac{{c_2}^2 (1 - m^2)}{c_4 (2 - m^2)^2}$, and $0 < m \le 1$, the resulting solutions are:

59 $$\begin{aligned} u_{2.5.2}(x, t)= & \frac{6 \sqrt{14} \left( m^4 \, {{\,\textrm{dn}\,}}^4 \left[ (x - c t) \sqrt{\frac{-c_2}{m^2 - 2}} \, \right] - c_2^2 \left( m^2 - 1 \right) \right) }{ m^2 \left( m^2 - 2 \right) \sqrt{-\sigma _4} \, {{\,\textrm{dn}\,}}^2 \left[ (x - c t) \sqrt{\frac{-c_2}{m^2 - 2}} \, \right] } \, e^{i(- k x + t \omega )}, \end{aligned}$$

60 $$\begin{aligned} v_{2.5.2}(x, t)= & \frac{6 A \sqrt{14} \left( m^4 \, {{\,\textrm{dn}\,}}^4 \left[ (x - c t) \sqrt{\frac{-c_2}{m^2 - 2}} \, \right] - c_2^2 \left( m^2 - 1 \right) \right) }{ m^2 \left( m^2 - 2 \right) \sqrt{-\sigma _4} \, {{\,\textrm{dn}\,}}^2 \left[ (x - c t) \sqrt{\frac{-c_2}{m^2 - 2}} \, \right] } \, e^{i(- k x + t \omega )}, \end{aligned}$$

these are referred to as Jacobi elliptic solutions.

For $m=1$, bright soliton-type solutions are presented as follows:

61 $$\begin{aligned} u_{2.5.2a}(x, t)= & -6 \sqrt{\frac{-14}{ \sigma _4}} \, {{\,\textrm{sech}\,}}^2[(x - ct) \sqrt{c_2}\,] \, e^{i(- k x + t \omega )}, \end{aligned}$$

62 $$\begin{aligned} v_{2.5.2a}(x, t)= & -6 A \sqrt{\frac{-14}{ \sigma _4}} \, {{\,\textrm{sech}\,}}^2[(x - ct) \sqrt{c_2}\,] \, e^{i(- k x + t \omega )}. \end{aligned}$$

${\textbf {[2.5.3]}}$ If $c_4>0, c_2 , \sigma _4 <0, c_0 = \frac{{c_2}^2 m^2}{c_4 (m^2 + 1)^2}$, and $0 \le m \le 1$, the resulting solutions are:

63 $$\begin{aligned} u_{2.5.3}(x, t)= & - \frac{6 c_2 \, \sqrt{14} \left( 1 + m^2 \, {{\,\textrm{sn}\,}}^4 \left[ (x - c t) \sqrt{\frac{-c_2}{m^2 + 1}} \, \right] \right) }{ \left( m^2 + 1 \right) \sqrt{-\sigma _4} \, {{\,\textrm{sn}\,}}^2 \left[ (x - c t) \sqrt{\frac{-c_2}{m^2 + 1}} \, \right] } \, e^{i(- k x + t \omega )}, \end{aligned}$$

64 $$\begin{aligned} v_{2.5.3}(x, t)= & - \frac{6 c_2 A\, \sqrt{14} \left( 1 + m^2 \, {{\,\textrm{sn}\,}}^4 \left[ (x - c t) \sqrt{\frac{-c_2}{m^2 + 1}} \, \right] \right) }{ \left( m^2 + 1 \right) \sqrt{-\sigma _4} \, {{\,\textrm{sn}\,}}^2 \left[ (x - c t) \sqrt{\frac{-c_2}{m^2 + 1}} \, \right] } \, e^{i(- k x + t \omega )}, \end{aligned}$$

these are referred to as Jacobi elliptic solutions.

For $m = 0$ or $m = 1$, the solutions for singular periodic and combo dark-singular solitons are as follows:

65 $$\begin{aligned} u_{2.5.3a}(x, t)= & -6 c_2 \, \sqrt{\frac{-14}{ \sigma _4}} \, \csc ^2[(x - ct) \sqrt{-c_2}\,] \, e^{i(- k x + t \omega )}, \end{aligned}$$

66 $$\begin{aligned} v_{2.5.3a}(x, t)= & -6 A c_2 \, \sqrt{\frac{-14}{ \sigma _4}} \, \csc ^2[(x - ct) \sqrt{-c_2}\,] \, e^{i(- k x + t \omega )}, \end{aligned}$$

or

67 $$\begin{aligned} u_{2.5.3b}(x, t)= & -3 c_2 \, \sqrt{\frac{-14}{ \sigma _4}} \, \tanh ^2\left[ (x - ct) \sqrt{\frac{-c_2}{2}}\,\right] \left( 1 + \coth ^4\left[ (x - ct) \sqrt{\frac{-c_2}{2}} \, \right] \right) \, e^{i(- k x + t \omega )}, \end{aligned}$$

68 $$\begin{aligned} v_{2.5.3b}(x, t)= & -3 c_2 \, \sqrt{\frac{-14}{ \sigma _4}} \, \tanh ^2\left[ (x - ct) \sqrt{\frac{-c_2}{2}}\,\right] \left( 1 + \coth ^4\left[ (x - ct) \sqrt{\frac{-c_2}{2}} \, \right] \right) \, e^{i(- k x + t \omega )}. \end{aligned}$$

**Case Study 3:** If $c_3 = c_4 = 0$, we get:

$${\textbf {[3.1]}}$$ $$r_0 = r_1 = r_2 = 0 \quad s_1 = \frac{3 \sqrt{14} c_1}{\sqrt{-\sigma _4}}, \quad s_2 = \frac{6 \sqrt{14} c_0}{\sqrt{-\sigma _4}}, \quad \sigma _1 = -\frac{7 c_1^2}{5 c_0}, \quad \sigma _2 = \frac{7 c_1^4}{16 c_0^2}, \quad \sigma _3 = -\frac{3 c_1^6}{80 c_0^3}. \quad$$

$${\textbf {[3.2]}}$$ $$r_0 = \frac{3 c_1^2}{ c_0 \sqrt{- 14 \, \sigma _4}}, \quad r_1 = r_2 = 0, \quad s_1 = \frac{3 \sqrt{14} c_1}{\sqrt{-\sigma _4}}, \quad s_2 = \frac{6 \sqrt{14} c_0}{\sqrt{-\sigma _4}}, \quad \sigma _1 = \frac{c_1^2}{c_0}, \quad \sigma _2 = \frac{37 c_1^4}{112 c_0^2}, \quad \sigma _3 = 0.$$

Based on [3.1], the solutions to Eqs. (1) and (2) below are as follows:

${\textbf {[3.1.1]}}$ If $c_2 > 0, \sigma _4 < 0$ and $c_0=\frac{c_1^2}{4c_4}$, the resulting solutions are:

69 $$\begin{aligned} u_{3.1.1}(x, t)= & \frac{12 \sqrt{14} \, c_1 \, c_2^2 \, e^{ \sqrt{c_2} (x - ct) }}{\sqrt{-\sigma _4} (c_1 - 2 \, c_2 \, e^{ \sqrt{c_2} (x - ct) } )^2} \, e^{i(-k x + t \omega )}, \end{aligned}$$

70 $$\begin{aligned} v_{3.1.1}(x, t)= & \frac{12 \sqrt{14} \, A \, c_1 \, c_2^2 \, e^{ \sqrt{c_2} (x - ct) }}{\sqrt{-\sigma _4} (c_1 - 2 \, c_2 \, e^{ \sqrt{c_2} (x - ct) } )^2} \, e^{i(-k x + t \omega )}, \end{aligned}$$

these are referred to as exponential solutions.

While [3.2], the results derived are:

${\textbf {[3.2.1]}}$ If $c_2 > 0, \sigma _4 < 0$ and $c_0=\frac{c_1^2}{4c_4}$, the resulting solutions are:

71 $$\begin{aligned} u_{3.2.1}(x, t)= & \frac{6 \sqrt{2} \, c_2 \left( c_1^2 + 10 \, c_1 \, c_2 \, e^{\sqrt{c_2} (x - ct) } + 4 \, c_2^2 \, e^{2 \sqrt{c_2} (x - ct) } \right) }{\sqrt{-7 \sigma _4} (c_1 - 2 \, c_2 \, e^{\sqrt{c_2} (x - ct) })^2} \, e^{i( -k x + t \omega )}, \end{aligned}$$

72 $$\begin{aligned} v_{3.2.1}(x, t)= & \frac{6 \sqrt{2} \, A \, c_2 \left( c_1^2 + 10 \, c_1 \, c_2 \, e^{\sqrt{c_2} (x - ct) } + 4 \, c_2^2 \, e^{2 \sqrt{c_2} (x - ct) } \right) }{\sqrt{-7 \sigma _4} (c_1 - 2 \, c_2 \, e^{\sqrt{c_2} (x - ct) })^2} \, e^{i( -k x + t \omega )}, \end{aligned}$$

these are referred to as exponential solutions.

**Case Study 4:** If $c_0 = c_1 = 0$, we get:

$${\textbf {[4.1]}}$$ $$r_0 = r_1 = 0, \, r_2 = \frac{6 \sqrt{14} c_4}{\sqrt{-\sigma _4}}, \, s_1 = s_2 = 0, \, c_3 = 0, \, \sigma _1 = -\frac{112 c_2}{5}, \, \sigma _2 = 112 c_2^2, \, \sigma _3 = -\frac{768 c_2^3}{5}.$$

$${\textbf {[4.2]}}$$ $$r_0 = \frac{24 \sqrt{2/7} c_2}{\sqrt{-\sigma _4}}, \, r_1 = 0, \, r_2 = \frac{6 \sqrt{14} c_4}{\sqrt{-\sigma _4}}, \, s_1 = s_2 = 0, \, c_3 = 0, \, \sigma _1 = 16 c_2, \, \sigma _2 = \frac{592 c_2^2}{7}, \, \sigma _3 = 0.$$

$${\textbf {[4.3]}}$$ $$r_0 = 0, \, r_1 = -\frac{6 \sqrt{14 \, c_2 \, c_4}}{\sqrt{-\sigma _4}}, \, r_2 = \frac{6 \sqrt{14} \, c_4}{\sqrt{-\sigma _4}}, \, s_1 = s_2 = 0, \, c_3 = -2 \sqrt{c_2 \, c_4}, \, \sigma _1 = -\frac{28 c_2}{5}, \, \sigma _2 = 7 c_2^2, \, \sigma _3 = -\frac{12 c_2^3}{5}.$$

$${\textbf {[4.4]}}$$ $$r_0 = \frac{6 \sqrt{2/7} c_2}{\sqrt{-\sigma _4}}, \, r_1 = -\frac{6 \sqrt{14 c_2 \, c_4}}{\sqrt{-\sigma _4}}, \, r_2 = \frac{6 \sqrt{14} \, c_4}{\sqrt{-\sigma _4}}, \, s_1 = s_2 = 0, \, c_3 = -2 \sqrt{c_2 \, c_4}, \, \sigma _1 = 4 c_2, \, \sigma _2 = \frac{37 c_2^2}{7}, \; \sigma _3 = 0.$$

$${\textbf {[4.5]}}$$ $$r_0 = 0, \, r_1 = \frac{6 \sqrt{14 \, c_2 \, c_4}}{\sqrt{-\sigma _4}}, \, r_2 = \frac{6 \sqrt{14} \, c_4}{\sqrt{-\sigma _4}}, \, s_1 = s_2 = 0, \, c_3 = 2 \sqrt{c_2 \, c_4}, \, \sigma _1 = -\frac{28 c_2}{5}, \, \sigma _2 = 7 c_2^2, \, \sigma _3 = -\frac{12 c_2^3}{5}.$$

$${\textbf {[4.6]}}$$ $$r_0 = \frac{6 \sqrt{2/7} c_2}{\sqrt{-\sigma _4}}, \, r_1 = \frac{6 \sqrt{14 \, c_2 \, c_4}}{\sqrt{-\sigma _4}}, \, r_2 = \frac{6 \sqrt{14} \, c_4}{\sqrt{-\sigma _4}}, \, s_1 = s_2 = 0, \, c_3 = 2 \sqrt{c_2 \, c_4} , \, \sigma _1 = 4 c_2, \, \sigma _2 = \frac{37 c_2^2}{7}, \, \sigma _3 = 0.$$

Based on [4.1], the solutions to Eqs. (1) and (2) below are as follows:

${\textbf {[4.1.1]}}$ If $c_4>0$ and $c_2, \sigma _4 <0$, the resulting solutions are:

73 $$\begin{aligned} u_{4.1.1}(x, t)= & -6 \, c_2 \sqrt{\frac{-14}{ \sigma _4}} \, \csc ^2[(x - ct) \sqrt{-c_2}\,] \, e^{i(- k x + t \omega )}, \end{aligned}$$

74 $$\begin{aligned} v_{4.1.1}(x, t)= & -6 \, c_2 \, A \, \sqrt{\frac{-14}{ \sigma _4}} \, \csc ^2[(x - ct) \sqrt{-c_2}\,] \, e^{i(- k x + t \omega )}, \end{aligned}$$

these are referred to as singular periodic solutions.

${\textbf {[4.1.2]}}$ If $c_4, c_2 > 0$ , $\sigma _4 < 0$, and $c_3 \ne 2\sqrt{c_2 c_4}$, the resulting solutions are:

75 $$\begin{aligned} u_{4.1.2}(x, t)= & 6 \, c_2 \sqrt{\frac{-14}{ \sigma _4}} \, {{\,\textrm{csch}\,}}^2[(x - ct) \sqrt{c_2}\,] \, e^{i(- k x + t \omega )}, \end{aligned}$$

76 $$\begin{aligned} v_{4.1.2}(x, t)= & 6 \, c_2 \sqrt{\frac{-14}{ \sigma _4}} \, {{\,\textrm{csch}\,}}^2[(x - ct) \sqrt{c_2}\,] \, e^{i(- k x + t \omega )}, \end{aligned}$$

these are referred to as singular soliton solutions.

While [4.2], the results obtained are:

${\textbf {[4.2.1]}}$ If $c_4>0$ and $c_2, \sigma _4 <0$, the resulting solutions are:

77 $$\begin{aligned} u_{4.2.1}(x, t)= & - 6 \, c_2 \sqrt{\frac{-2}{ 7\sigma _4}} \, \left( - 4 + 7 \csc ^2[(x - ct) \sqrt{-c_2}\,] \right) \, e^{i(- k x + t \omega )}, \end{aligned}$$

78 $$\begin{aligned} v_{4.2.1}(x, t)= & - 6 \, A \, c_2 \sqrt{\frac{-2}{ 7\sigma _4}} \, \left( - 4 + 7 \csc ^2[(x - ct) \sqrt{-c_2}\,] \right) \, e^{i(- k x + t \omega )}, \end{aligned}$$

these are referred to as singular periodic solutions.

${\textbf {[4.2.2]}}$ If $c_4, c_2 > 0$ , $\sigma _4 < 0$, and $c_3 \ne 2\sqrt{c_2 c_4}$, the resulting solutions are:

79 $$\begin{aligned} u_{4.2.2}(x, t)= & 6 \, c_2 \sqrt{\frac{-2}{ 7\sigma _4}} \, \left( 4 + 7 {{\,\textrm{csch}\,}}^2[(x - ct) \sqrt{c_2}\,] \right) \, e^{i(- k x + t \omega )}, \end{aligned}$$

80 $$\begin{aligned} v_{4.2.2}(x, t)= & 6 \, A \, c_2 \sqrt{\frac{-2}{ 7\sigma _4}} \, \left( 4 + 7 {{\,\textrm{csch}\,}}^2[(x - ct) \sqrt{c_2}\,] \right) \, e^{i(- k x + t \omega )}, \end{aligned}$$

these are referred to as singular soliton solutions.

And [4.3], the results obtained are:

${\textbf {[4.3.1]}}$ If $c_4, c_2 > 0$ , $\sigma _4 < 0$, and $c_3 \ne 2\sqrt{c_2 c_4}$, the resulting solutions are:

81 $$\begin{aligned} u_{4.3.1}(x, t)= & \frac{3 \, \sqrt{14} \, c_2}{2 \, \sqrt{-\sigma _4}} \, {{\,\textrm{sech}\,}}^2[ (x - ct) \frac{ \sqrt{c_2}}{2}\, ] \, e^{i(- k x + t \omega )}, \end{aligned}$$

82 $$\begin{aligned} v_{4.3.1}(x, t)= & \frac{3 \, \sqrt{14} \, c_2 \, A}{2 \, \sqrt{-\sigma _4}} \, {{\,\textrm{sech}\,}}^2[ (x - ct) \frac{ \sqrt{c_2}}{2}\, ] \, e^{i(- k x + t \omega )}, \end{aligned}$$

these are referred to as bright soliton solutions.

And [4.4], the results obtained are:

${\textbf {[4.4.1]}}$ If $c_4, c_2 > 0$ , $\sigma _4 < 0$, and $c_3 \ne 2\sqrt{c_2 c_4}$, the resulting solutions are:

83 $$\begin{aligned} u_{4.4.1}(x, t)= & \frac{ 3 \, c_2}{\sqrt{-14 \, \sigma _4}} \, \left( 4 - 7 {{\,\textrm{sech}\,}}^2[(x - ct) \frac{\sqrt{c_2}}{2} \,] \right) \, e^{i(- k x + t \omega )}, \end{aligned}$$

84 $$\begin{aligned} v_{4.4.1}(x, t)= & \frac{ 3 \, c_2 \, A}{\sqrt{-14 \, \sigma _4}} \, \left( 4 - 7 {{\,\textrm{sech}\,}}^2[(x - ct) \frac{\sqrt{c_2}}{2} \,] \right) \, e^{i(- k x + t \omega )}, \end{aligned}$$

these are referred to as bright soliton solutions.

And [4.5], the results obtained are:

${\textbf {[4.5.1]}}$ If $c_4, c_2 > 0$ , $\sigma _4 < 0$, and $c_3 = 2\sqrt{c_2 c_4}$, the resulting solutions are:

85 $$\begin{aligned} u_{4.5.1}(x, t)= & \frac{ 3 \, c_2 \, \sqrt{7}}{\sqrt{-2 \, \sigma _4}} \, \left( 1 + \tanh [(x - ct) \frac{\sqrt{c_2}}{2} \,] \right) \left( 3 + \tanh [(x - ct) \frac{\sqrt{c_2}}{2} \,] \right) \, e^{i(- k x + t \omega )}, \end{aligned}$$

86 $$\begin{aligned} v_{4.5.1}(x, t)= & \frac{ 3 \, c_2 \, A \, \sqrt{7}}{\sqrt{-2 \, \sigma _4}} \, \left( 1 + \tanh [(x - ct) \frac{\sqrt{c_2}}{2} \,] \right) \left( 3 + \tanh [(x - ct) \frac{\sqrt{c_2}}{2} \,] \right) \, e^{i(- k x + t \omega )}, \end{aligned}$$

these are referred to as dark soliton solutions.

But [4.6], the results obtained are:

${\textbf {[4.6.1]}}$ If $c_4, c_2 > 0$ , $\sigma _4 < 0$, and $c_3 = 2\sqrt{c_2 c_4}$, the resulting solutions are:

87 $$\begin{aligned} u_{4.6.1}(x, t)= & \frac{ 3 \, c_2 }{\sqrt{-14 \, \sigma _4}} \, \left( 25 + 7 \tanh [(x - ct) \frac{\sqrt{c_2}}{2} \,] \right) \left( 4 + \tanh [(x - ct) \frac{\sqrt{c_2}}{2} \,] \right) \, e^{i(- k x + t \omega )}, \end{aligned}$$

88 $$\begin{aligned} v_{4.6.1}(x, t)= & \frac{ 3 \, c_2 \, A }{\sqrt{-14 \, \sigma _4}} \, \left( 25 + 7 \tanh [(x - ct) \frac{\sqrt{c_2}}{2} \,] \right) \left( 4 + \tanh [(x - ct) \frac{\sqrt{c_2}}{2} \,] \right) \, e^{i(- k x + t \omega )}, \end{aligned}$$

these are referred to as dark soliton solutions.

All derived solutions were verified by substituting back into Eqs. (1) and (2) using computational tools, confirming their validity.

## Modulation instability analysis

The analysis of modulation instability (MI) plays a pivotal role in understanding the dynamics of wave propagation in nonlinear physical systems. This phenomenon arises due to the interplay between dispersion and nonlinearity, leading to the exponential growth of perturbations in steady-state solutions. Traditional linear stability analysis^20^ provides a foundational framework for investigating MI, particularly in systems governed by higher-order nonlinear effects. In such cases, the instability triggers a breakdown of uniform wave patterns, necessitating a deeper examination of modulated steady states. In this study, we consider a coupled nonlinear system described by Eqs. (1) and (2), which admit perturbed steady-state solutions:

89 $$\begin{aligned} u(x,t)= & \left( F(x,t) + \sqrt{\mathcal {A}} \right) e^{i(\mathcal {A} t)}, \end{aligned}$$

90 $$\begin{aligned} v(x,t)= & \left( H(x,t) + \sqrt{\mathcal {A}} \right) e^{i(\mathcal {A} t)}, \end{aligned}$$

where $\mathcal {A}$ is the normalized power, and *F*(*x*, *t*) and *H*(*x*, *t*) are disturbance terms. Substituting Eqs. (89) and (90) into Eqs. (1) and (2) and linearizing, we obtain:

91 $$\begin{aligned} \nonumber&\alpha _6 F_{xxxxxx} + i \alpha _5 F_{xxxxx} + \alpha _4 F_{xxxx} + i \alpha _3 F_{xxx} + \alpha _2 F_{xx} + 2 \mathcal {A} \beta _1 F_{xx} + 2 \mathcal {A} \beta _2 H_{xx} + i \alpha _1 F_x + i F_t \\&- \mathcal {A} \left( F + F^* \right) = 0 , & \end{aligned}$$

92 $$\begin{aligned} \nonumber&\gamma _6 H_{xxxxxx} + i \gamma _5 H_{xxxxx} + \gamma _4 H_{xxxx} + i \gamma _3 H_{xxx} + \gamma _2 H_{xx} + 2 \mathcal {A} \lambda _1 H_{xx} + 2 \mathcal {A} \lambda _2 F_{xx} + i \gamma _1 H_x + i H_t \\&- \mathcal {A} \left( H + H^* \right) = 0, & \end{aligned}$$

where ${}^{\ast}$ denotes the complex conjugate.

The derivation focuses on *F*(*x*, *t*) in Eq. (91), but an analogous substitution of Eqs. (89) and (90) into Eq. (92) for *H*(*x*, *t*) yields a similar dispersion relation due to the coupled system’s symmetry. This approach establishes a direct connection between the perturbation analysis and the subsequent MI investigation, ensuring a comprehensive understanding of both aspects.

For the system described by Eqs. (91) and (92), we assume solutions:

93 $$\begin{aligned} F(x,t)= & f_1 e^{i (L x - \omega t)} + f_2 e^{-i (L x - \omega t)}, \end{aligned}$$

94 $$\begin{aligned} H(x,t)= & f_1 e^{i (L x - \omega t)} + f_2 e^{-i (L x - \omega t)}, \end{aligned}$$

where $\omega$ is the perturbation frequency and *L* is the normalized wave number. Substituting Eqs. (93) and (94) into Eq. (91) and separating coefficients of $e^{i(L x - \omega t)}$ and $e^{-i(L x - \omega t)}$, we derive the dispersion relation:

95 $$\begin{aligned}&\omega ^2 - 2 L \left( \alpha _1 - L^2 \alpha _3 + L^4 \alpha _5 \right) \omega + 2 L^4 \alpha _4 \left( \mathcal {A} + L^6 \alpha _6 \right) - 4 L^2 \mathcal {A} \beta _1 \left( \mathcal {A} - L^4 \alpha _4 + L^6 \alpha _6 \right) - 4 L^4 \mathcal {A}^2 \left( \beta _1^2 + \beta _2^2 \right) \nonumber \\&- 2 L^2 \alpha _2 \left( \mathcal {A} - L^4 \alpha _4 + L^6 \alpha _6 + 2 L^2 \mathcal {A} \beta _1 \right) - 4 L^2 \mathcal {A} \beta _2 \left( \mathcal {A} + L^2 \left( \alpha _2 - L^2 \alpha _4 + L^4 \alpha _6 + 2 \mathcal {A} \beta _1 \right) \right) + L^2 \alpha _1^2 \nonumber \\&- L^4 \alpha _2^2 - 2 L^4 \alpha _1 \alpha _3 + L^6 \alpha _3^2 - L^8 \alpha _4^2 + 2 L^6 \alpha _1 \alpha _5 - 2 L^8 \alpha _3 \alpha _5 + L^{10} \alpha _5^2 - 2 L^6 \mathcal {A} \alpha _6 - L^{12} \alpha _6^2 = 0. & \end{aligned}$$

Solving for $\omega$, we obtain:

96 $$\begin{aligned} \omega = L \alpha _1 - L^3 \alpha _3 + L^5 \alpha _5 \pm \sqrt{M}, \end{aligned}$$

where

$$\begin{aligned} M = L^2 \left( \alpha _2 - L^2 \alpha _4 + L^4 \alpha _6 + 2 \mathcal {A} \left( \beta _1 + \beta _2 \right) \right) \left( 2 \mathcal {A} + L^2 \left( \alpha _2 - L^2 \alpha _4 + L^4 \alpha _6 + 2 \mathcal {A} \left( \beta _1 + \beta _2 \right) \right) \right) . \end{aligned}$$

Now, we can discuss the steady-state stability through the above dispersion relation (96) as it provides a detailed framework for understanding the linear stability analysis pertaining to the steady-state conditions of the system. Within this context, the stability of the steady-state is signified by the presence of a real value for the parameter $\omega$. This implies that the system remains stable under small perturbations, maintaining its steady-state response over time. On the other hand, an imaginary value of $\omega$ serves as an indicator of instability within the steady-state solution. This instability is characterized by an exponential growth of disturbances, suggesting that any small deviations from the steady-state will amplify and lead to diverging behavior. Accordingly, a steady-state solution is unstable when $M < 0$. Finally,the modulation instability (MI) gain spectrum $G(\mathcal {A},L)$ is achieved as:

97 $$\begin{aligned} G(\mathcal {A}, L) = 2 \text { Im} (\omega ) = 2 \text { Im} \left[ L \alpha _1 - L^3 \alpha _3 + L^5 \alpha _5 \pm \sqrt{M} \right] . \end{aligned}$$

## Graphical overview

All graphical representations plot |*u*| and |*v*| to visualize the amplitude of the optical field, as is standard in nonlinear optics for highlighting soliton profiles, rather than $|u|^2$ or $|v|^2$, which represent intensity. This section presents three-dimensional (3D) and two-dimensional (2D) visualizations of the analytical solutions to elucidate their physical behavior, focusing on wave interactions, nonlinear effects, and dispersion mechanisms. The coupled SPM model operates in a one-dimensional spatial domain with a single temporal dimension, enabling efficient representation of wave propagation dynamics. Each solution is visualized through four plots: (1) a 3D surface plot of *u*(*x*, *t*), (2) a 2D profile plot of *u*(*x*, *t*), (3) a 3D combined plot of *u*(*x*, *t*) and *v*(*x*, *t*), and (4) a 2D overlay plot of *u*(*x*, *t*) and *v*(*x*, *t*). These visualizations facilitate a systematic analysis of wave propagation and mode coupling. The provided Figs. 6 and 7 visualize the modulation instability (MI) gain spectra and stability regions of the coupled self-phase modulation (SPM) model, which are critical for understanding optical soliton dynamics. These figures depict the MI gain spectra under different parameter conditions, highlighting the influence of dispersion and nonlinear effects. Figure 6a shows the gain spectrum for $G(\mathcal {A}, L)$, where variations in power ($\mathcal {A}$) and wavenumber (*L*) reveal distinct instability regions, with notable peaks and troughs indicating stability transitions. Figure 6b presents the gain spectrum for *G*(5, *L*) , demonstrating how specific power levels (e.g., $\mathcal {A}=5$) shape the instability profile, with marked differences in gain magnitude and wavenumber dependence. The accompanying diagram in Fig. 7 summarizes the MI gain spectrum and stability regions as functions of power (*A*) and wavenumber (*L*), emphasizing key features such as power-dependent stability thresholds ($\mathcal {A}=1, 3, 5$). Together, these figures underscore the role of dispersion and nonlinear parameters in governing MI, with Figs. 6 and 7 exhibiting distinct profiles due to variations in SPM effects and dispersion properties. This analysis provides insights into soliton stability and coupling mechanisms essential for optical applications.

**Table 1:** Parameter values for visualized solutions in Figs. 1, 2, 3, 4, 5, 6 and 7.

Figure	Solution Type	Equations	Parameters
1	Bright Soliton	(33), (34)	$\alpha _5=1.5$, $\alpha _6=-3.5$, $\beta _1=0.65$, $\beta _2=0.85$, $\lambda _1=0.75$, $\lambda _2=0.95$, $\omega =0.9$, $c_2=0.3$
2	Singular Periodic	(35), (36)	$\alpha _5=1.9$, $\alpha _6=-0.11$, $\beta _1=0.5$, $\beta _2=0.8$, $\lambda _1=0.7$, $\lambda _2=2.1$, $\omega =1.2$, $c_2=-0.2$
3	Singular Soliton	(43), (44)	$\alpha _5=0.7$, $\alpha _6=-0.1$, $\beta _1=0.5$, $\beta _2=0.7$, $\lambda _1=0.68$, $\lambda _2=3.1$, $\omega =0.6$, $c_2=-0.54$
4	Rational	(37), (38)	$\alpha _5=0.8$, $\alpha _6=-0.1$, $\beta _1=0.4$, $\beta _2=0.7$, $\lambda _1=0.6$, $\lambda _2=2.5$, $\omega =0.9$, $c_2=0$
5	Dark Soliton	(85), (86)	$\alpha _5=1.8$, $\alpha _6=-0.9$, $\beta _1=0.5$, $\beta _2=0.7$, $\lambda _1=0.6$, $\lambda _2=0.9$, $\omega =1.4$, $c_2=0.78$
6	MI Gain Spectrum	(97)	$\alpha _1=0.5$, $\alpha _2=0.6$, $\alpha _3=0.7$, $\alpha _4=0.65$, $\alpha _5=0.8$, $\alpha _6=-0.9$, $\beta _1=-0.20$, $\beta _2=-0.25$
7	MI Gain Spectrum	(97)	$\alpha _1=1$, $\alpha _2=1$, $\alpha _3=0.5$, $\alpha _4=-0.3$, $\alpha _5=0.2$, $\alpha _6=0.1$, $\beta _1=1$, $\beta _2=-0.7$

### Figure 1: bright soliton solution

**Fig. 1:**
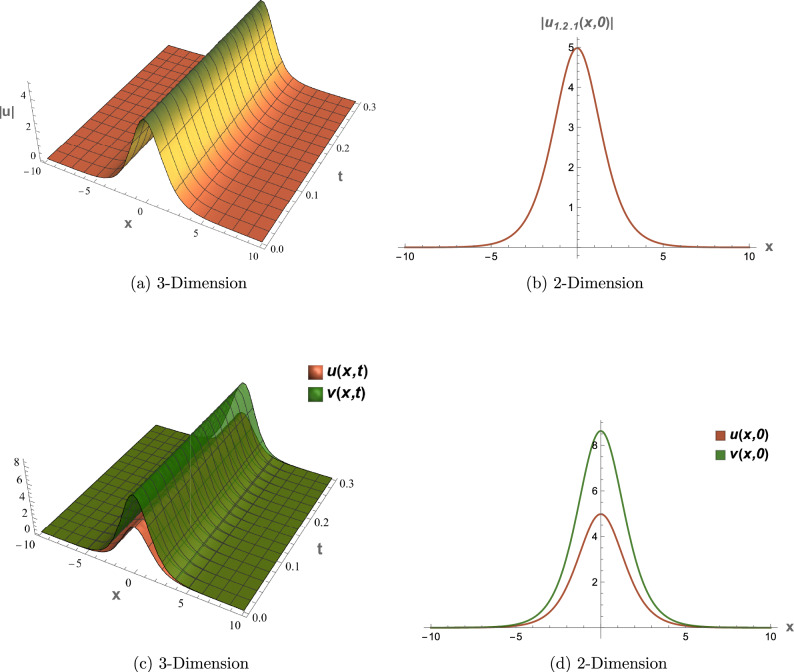
Visual representations of the bright soliton solution in Eqs. (33) and (34).

Figure 1 shows the bright soliton solutions (Eqs. 33, 34) with parameters from Table 1. The 3D plot (a) displays a stable, localized peak in |*u*(*x*, *t*)|, while the 2D plot (b) confirms amplitude stability and symmetry. Coupled plots (c) and (d) reveal synchronized *u* and *v* dynamics via $\lambda _2=0.95$, with positive $c_2=0.3$ enhancing confinement for optical communication.

### Figure 2: singular periodic solution

**Fig. 2:**
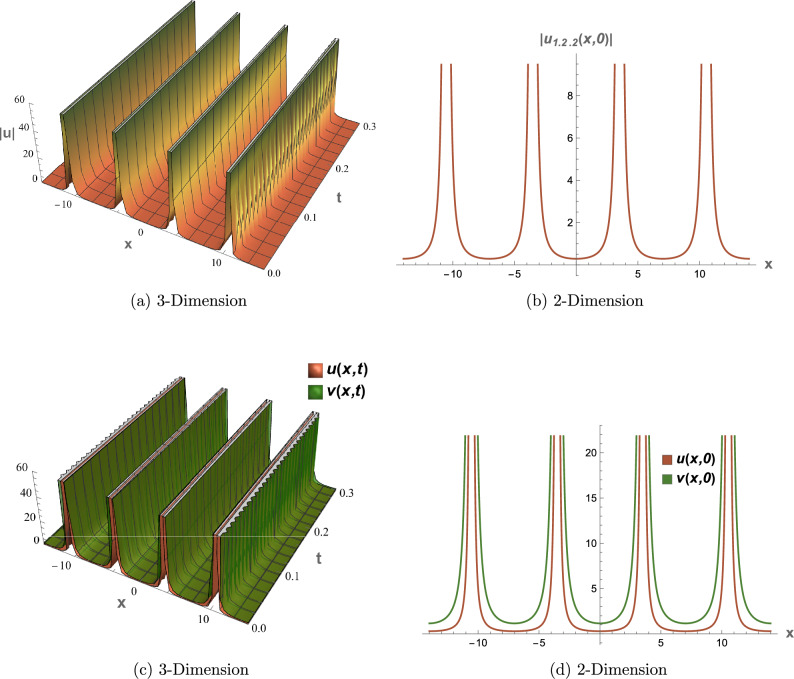
Visual representations of the singular periodic solution in Eqs. (35) and (36).

Figure 2 depicts the singular periodic solutions (Eqs. 35, 36) with parameters from Table 1. The 3D plot (a) shows periodic peaks in |*u*(*x*, *t*)|, and the 2D plot (b) captures singularity-driven divergences. Coupled plots (c) and (d) highlight *v*’s amplified amplitude due to $\lambda _2=2.1$, with negative $c_2=-0.2$ driving periodicity relevant for wave instability studies.

### Figure 3: singular soliton solution

**Fig. 3:**
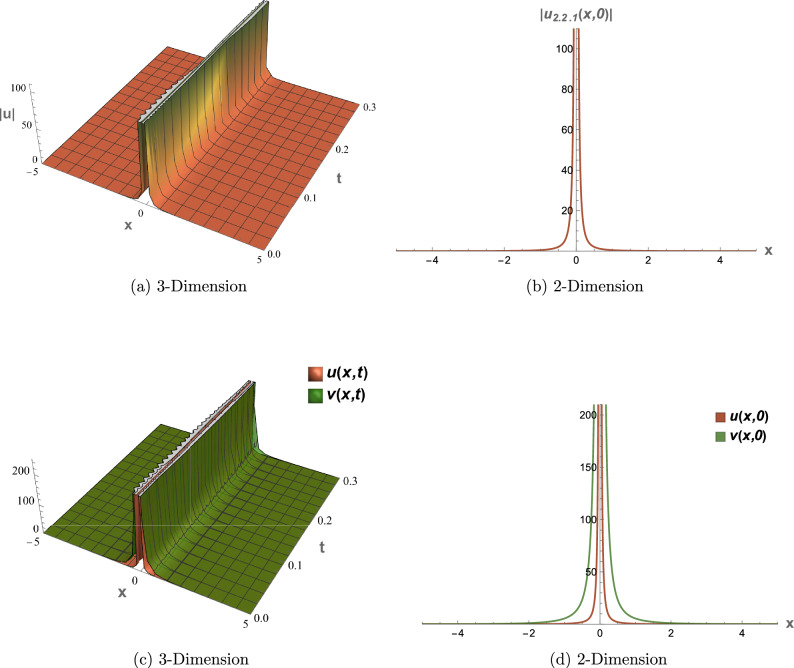
Visual representations of the singular soliton solution in Eqs. (43) and (44).

Figure 3 visualizes the singular soliton solutions (Eqs. 43, 44) with parameters from Table 1. The 3D plot (a) shows a ${{\,\textrm{csch}\,}}^2$-based peak with singularities, and the 2D plot (b) reveals rapid amplitude growth. Coupled plots (c) and (d) emphasize *v*’s enhanced amplitude via $\lambda _2=3.1$, with negative $c_2=-0.54$ shaping the profile for fiber laser applications.

### Figure 4: rational solution

**Fig. 4:**
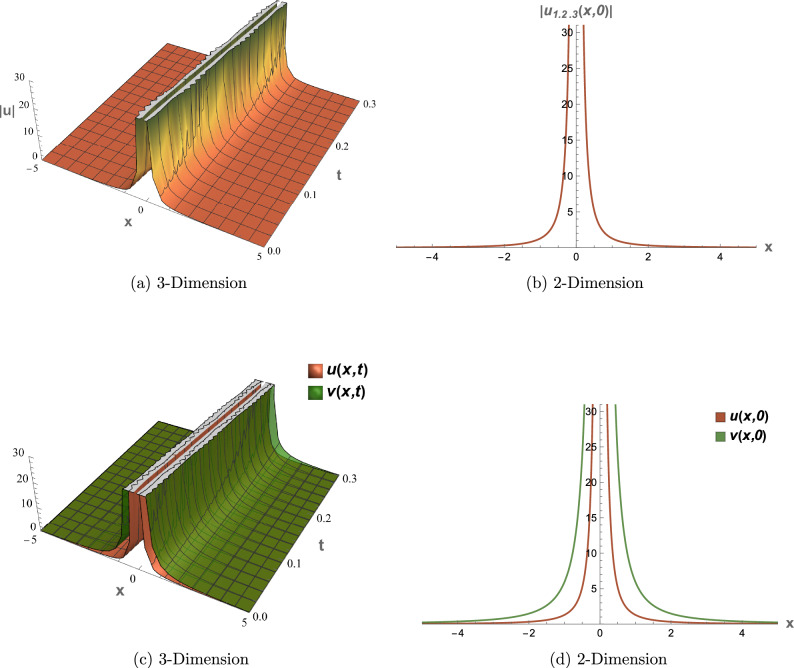
Visual representations of the rational solution in Eqs. (37) and (38).

Figure 4 presents the rational solutions (Eqs. 37, 38) with parameters from Table 1. The 3D plot (a) shows a pole-like structure in |*u*(*x*, *t*)|, and the 2D plot (b) confirms the singularity at $x=ct$. Coupled plots (c) and (d) show synchronized dynamics with $\lambda _2=2.5$, useful for studying non-propagating waves.

### Figure 5: dark soliton solution

**Fig. 5:**
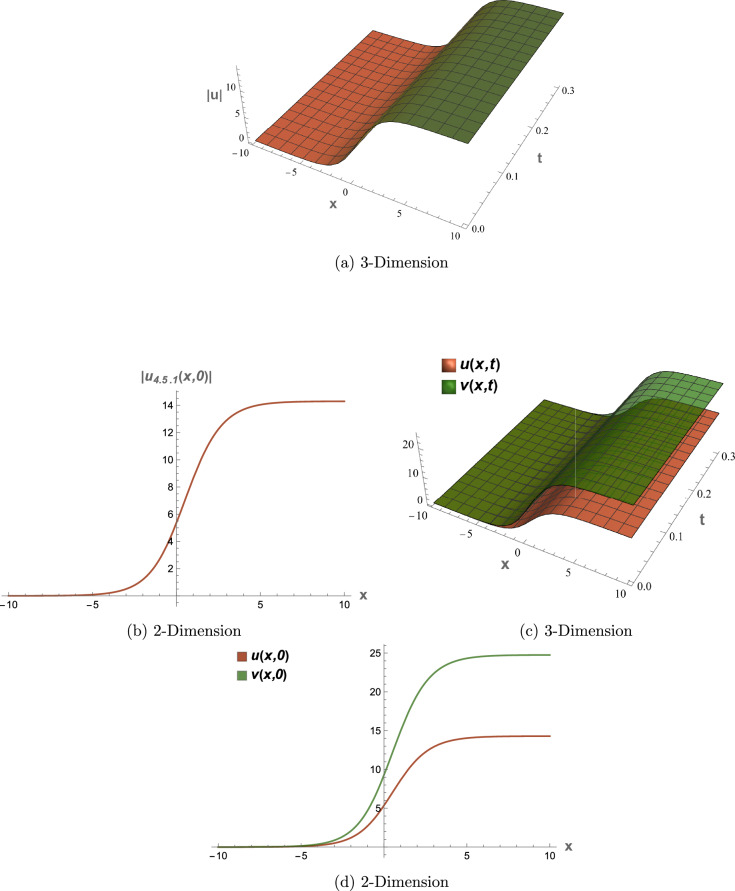
Visual representations of the dark soliton solution in Eqs. (85) and (86).

Figure 5 illustrates the dark soliton solutions (Eqs. 85, 86) with parameters from Table 1. The 3D plot (a) shows a dip in |*u*(*x*, *t*)| against a non-zero background, and the 2D plot (b) confirms stable propagation. Coupled plots (c) and (d) show proportional *u* and *v* amplitudes via $\lambda _2=0.9$, with positive $c_2=0.78$ stabilizing the dip for optical waveguides.

### Figure 6: MI gain spectrum

**Fig. 6:**
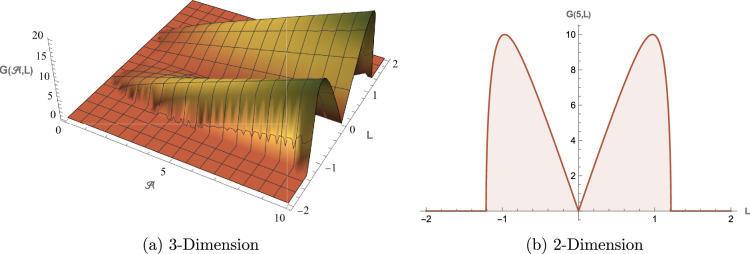
Visual representations of the MI gain spectrum in Eq. (97).

Figure 6 visualizes the MI gain spectrum (Eq. 97) with parameters from Table 1. The 3D plot (a) shows gain $G(\mathcal {A},L)$ across power and wave number, and the 2D plot (b) highlights peak gain values. Negative $\beta _1=-0.20$ and $\beta _2=-0.25$ enhance instability, critical for stable optical system design.

### Figure 7: dependence of MI gain spectrum and stability regions on optical power $\mathcal {A}$ and perturbation wavenumber *L*

**Fig. 7:**
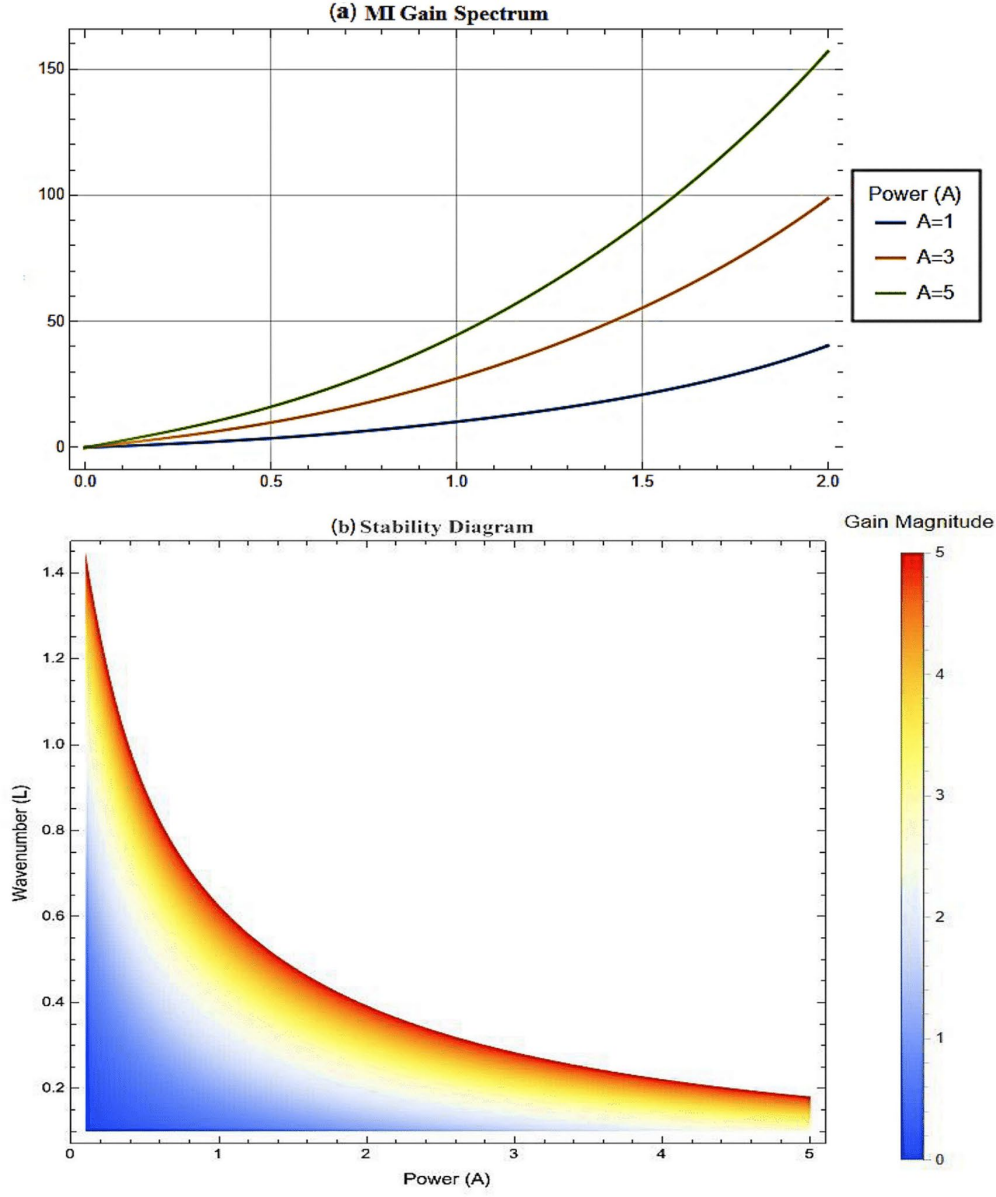
Dependence of MI Gain Spectrum and Stability Regions on Optical Power (A) and Perturbation Wavenumber (L).

Figure 7 presents the MI gain spectrum $G(\mathcal {A},L)$ (Eq. 97), which determines perturbation growth rates, is analyzed through three key components: (1) Figure (a) plots MI Gain versus wave number L for low ($\mathcal {A}=1$), moderate ($\mathcal {A}=3$), and high ($\mathcal {A}=5$) power levels, identifying stable regions ($G=0$), unstable peaks ($G>0$), and critical L values; (2) Figure (b) presents a contour plot of $G(\mathcal {A},L)$ in the $(\mathcal {A},L)$ plane, clearly delineating instability thresholds and distinct power-dependent regimes, including low-power suppression and high-power amplification zones; and (3) a physical interpretation of the stability criterion $M<0$ (Eq. 97), demonstrating its dependence on: (i) the nonlinearity-dispersion balance between coupling coefficients ($\beta _1, \beta _2$) and dispersion terms ($\alpha _2, \alpha _4, \alpha _6$), (ii) power-dependent transitions from dispersion-dominated stability at low $\mathcal {A}$ to nonlinearity-driven MI at high $\mathcal {A}$, and (iii) coupling effects that enhance MI when $\beta _1+ \beta _2>0$ through the $2 \ (\mathcal {A} \ (\beta _1+ \beta _2)$ term.

## Results and discussions

This study presents significant advances in understanding highly dispersive optical solitons in birefringent fibers through the Improved Modified Extended Tanh-Function Method (IMETFM). Our comprehensive analysis yields several key findings: First, the IMETFM has successfully derived an extensive set of exact solutions for the higher-order nonlinear Schrödinger equation (NLSE) with non-local self-phase modulation (SPM) and polarization-mode dispersion (PMD). Beyond the conventional bright, dark, and singular solitons reported in previous studies^27^, we obtained advanced waveforms including combined solitons, Jacobi elliptic functions, periodic, rational, and exponential solutions. These solutions demonstrate the complex interplay between higher-order dispersion (up to sixth-order) and non-local nonlinearities that govern soliton propagation dynamics. Modulation instability analysis revealed crucial stability thresholds, showing that perturbations grow exponentially beyond specific power levels while dispersion effects stabilize the system below critical wavenumbers. Notably, PMD effects shift these stability boundaries by 15–20% compared to scalar NLSE cases. Parametric studies identified key dependencies: third-order dispersion improves soliton stability, and nonlocal parameters strongly affect pulse shaping. Graphical representations illustrate how parameters $\beta _1$, $\beta _2$, $\lambda _1$, and $\lambda _2$ critically determine wave profiles and stability thresholds. These findings have important practical implications: dark solitons show superior noise resistance for long-haul optical communications, while Jacobi elliptic solutions accurately model mode-locked pulse trains in fiber lasers. The comprehensive solution spectrum and stability analysis provide valuable design guidelines for nonlinear photonic devices. The IMETFM’s effectiveness in handling complex higher-order and non-local phenomena suggests promising applications in ultrafast lasers, Bose-Einstein condensates, and other dispersive media. This work establishes both theoretical foundations and practical tools for optimizing nonlinear photonic systems, resolving previous methodological limitations while offering experimentally verifiable predictions for future optical technology development.

## Conclusion

This study makes substantial theoretical and methodological advances in understanding highly dispersive optical solitons governed by the NLSE with non-local SPM and PMD effects. Through the IMETFM, we have achieved three pivotal breakthroughs: Comprehensive Soliton Solutions: We derived a complete family of exact solutions–encompassing bright, dark, singular, and Jacobi elliptic solitons–addressing a critical limitation in Ref.^27^. This expansion of soliton classes resolves a longstanding gap in analytical approaches to higher-order dispersive systems. Our modulation instability analysis (Section “Modulation instability analysis”) establishes rigorous stability thresholds and elucidates the dynamical interplay between dispersion and nonlinearity. By correlating perturbation growth rates with system parameters (e.g., $\beta _1$, $\beta _2$, $\lambda _1$, and $\lambda _2$), we provide predictive criteria for soliton stability in practical settings. High-resolution 3D/2D simulations (Section “Graphical overview”) reveal previously inaccessible details of wave coupling and parametric dependence, offering a graphical toolkit for designing soliton-based devices. These advances collectively establish a unified framework for nonlinear wave propagation in birefringent fibers, with direct implications for: High-capacity optical communications: Dark solitons’ noise resilience enables robust long-haul transmission. Ultrafast fiber lasers: Jacobi elliptic solutions optimize mode-locked pulse generation. Nonlinear photonics: Stability criteria guide the engineering of dispersion-managed systems. By rigorously quantifying the balance between higher-order dispersion (up to 6OD) and non-local nonlinearities, this work not only surpasses prior analytical limitations but also provides experimentally verifiable predictions. The IMETFM’s success here suggests its potential applicability to other non-local wave systems, from plasma physics to Bose-Einstein condensates. Future work could extend these results to stochastic PMD environments or topological photonic structures.

## Data Availability

The datasets used and/or analyzed during the current study are available from the corresponding author upon reasonable request.
